# Potential of Pulsed Electric Fields for the preparation of Spanish dry-cured sausages

**DOI:** 10.1038/s41598-019-52464-3

**Published:** 2019-11-05

**Authors:** Leire Astráin-Redín, Javier Raso, Guillermo Cebrián, Ignacio Álvarez

**Affiliations:** Departamento de Producción Animal y Ciencia de los Alimentos. Tecnología de los Alimentos. Facultad de Veterinaria. Instituto Agroalimentario de Aragón– IA2 - (Universidad de Zaragoza-CITA), Zaragoza, Spain

**Keywords:** Chemical engineering, Biochemistry

## Abstract

The aim of this investigation was to lay the groundwork of the potential application of Pulsed Electric Fields (PEF) technology for accelerating the drying process of meat and meat products, and specifically in this work of Spanish dry-cured sausages “longaniza”. PEF treatments were applied to pork loin samples, and the influence of different PEF parameters on the process were evaluated. An optimal PEF treatment of 1 kV/cm, 200 μs of pulse width and 28 kJ/kg was determined as the most suitable to electroporate meat cells and to improve water transfer by achieving a water content reduction of 60.4% in treated-meat samples dried at 4 °C. The influence of PEF on meat drying rate was also studied on minced pork and the results showed that with a particle size of 4.0 mm higher drying rates were achieved. To validate the results, Spanish cured sausages were prepared from treated and untreated minced pork and stuffed into gauzes and natural pork casings at pilot plant scale. After the curing process, the application of PEF to sausages stuffed into gauze reduced the drying time from 17 to 9–10 days, a reduction of 41–47%, confirming the effects described at lab scale and the potential of PEF for accelerating the sausage-drying process.

## Introduction

The process of drying and maturing (curing) of meat products, and particularly of raw-cured sausages, involves a series of physical, chemical and microbiological transformations. These phenomena are thoroughly dependent on curing conditions which determine the characteristics of the final product. Thus, in order to obtain products with a more intense color, better flavor, and a superior rate of preservation, slow ripening is sought, which is achieved by applying low temperatures (5–15 °C). These low temperatures imply longer manufacturing times, extending to months in some cases^1^, what makes the product and economic turnover of the companies very slow. Therefore, any strategy that can help shorten this process while maintaining the sensorial quality of the final meat product would be of great interest. Pulsed Electric Fields (PEF) could represent a positive alternative in order to accelerate water transfer and reduce drying times^2,3^.

Pulsed Electric Field (PEF) treatment consists of the application of electric fields of high intensity (>0.1 kV/cm) and short duration (from milliseconds to microseconds) to a product placed between two electrodes^4^. The application of PEF treatments leads to the electroporation of the cell membranes. The eventual result is the formation of aqueous pathways (or electropores), leading to an increase in cellular membrane permeability to ions and other molecules^5^. PEF is now recognized as a promising non-thermal technology for food preservation, since it maintains food quality while inactivating vegetative bacteria and yeast cells^6^. In recent years, besides its use for pasteurizing liquid food products such as juices, milk, or liquid egg products, other applications have been increasingly investigated, particularly its potential for improving mass transfer: e.g. for increasing of the yield in juice and/or oil production processes, for extracting polyphenols during red wine maceration, and for accelerating the drying rate in vegetables, among others^7–9^. However, very few publications have dealt with this technology’s potential for improving meat production processes, and the existing ones have been mainly focused on food safety, tenderization, supercooling, and brining processes but not on meat drying or curing^2,3^. Furthermore, it should be noted that, obtained results have not been conclusive in some cases and for some applications. This might be due to the difficulty of studying the effect of PEF on meat, where, in addition to treatment parameters (electric field strength, pulse width, specific energy or treatment time), the effects of electroporation would also depend on many intrinsic factors of the matrix, such as the animal species, meat cut, the orientation of the fibers, previously applied cold or freezing treatments, etc.^2,10^.

As pointed out above, most studies of the effect of PEF on meat products have focused on its application to the acceleration of meat tenderization, since, at least theoretically, the application of PEF would result in muscle fiber electroporation, thereby increasing the mobility of intracellular components responsible for proteolysis. More precisely, the electroporation of muscle cells would facilitate the release of calcium ions necessary for protease activation, thereby accelerating the proteolytic process responsible for meat tenderization/maturation^11^.

On the other hand, few studies have evaluated the potential of PEF for the acceleration of mass transfer processes in meat. Toepfl and Heinz^12^ observed that applying a pretreatment of 3 kV/cm improved salt and nitrite diffusion processes in brined pork products, as subsequently corroborated by Mc Donnell *et al*.^13^. In the same study Toepfl and Heinz^12^ observed that applying a treatment of 3 kV/cm and 5 kJ/kg to pork shoulder increased the weight (water) loss by 25% after 350 h of drying (at 8 °C and 95% relative humidity), as compared to non-treated samples. Therefore, it seems that this technology might be very interesting for meat-curing procedures in which water transfer processes are involved, similarly to what has been described for other matrices^14–17^.

However, hardly any data is available regarding PEF-assisted meat curing, a process in which water mass transfer is one of the main mechanisms involved or the PEF conditions indicated in the literature not always result in the improvement of water transfer, and no data is available of the effect of PEF in minced meat used to prepare cured dry products. Therefore, the objective of this investigation was to do a detailed study of the effect of the main PEF treatment parameters to define the most adequate PEF treatment conditions to electroporate meat cells improving the water transfer, and based on these conditions to evaluate the influence of the application of PEF on pork meat drying rates and its potential for the acceleration of meat-curing processes, such as in Spanish raw-cured sausage manufacturing.

## Material and Methods

### Samples

In order to evaluate the influence of PEF on drying processes, pork loins of Duroc breed purchased at a local supermarket were used. The average electrical conductivity of the samples measured with an impedanciometer (DIL – DEUTSCHES INSTITUT FÜR LEBENSMITTELTECHNIK E.V., Quakenbrück, Germany) was 5.6 ± 0.1 mS/cm and the specific heat, 2.8 kJ/kg °C^18^.

**Figure 1:**
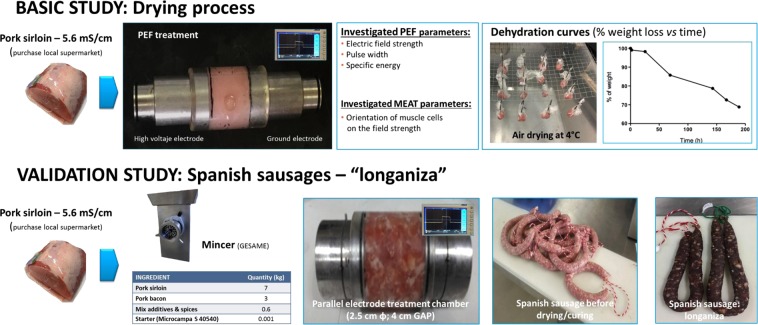
Experimental procedures followed in the work.

In a second set of experiments designed to validate the results obtained with pork loin, the drying of Spanish dry-cured sausage (“longaniza”) assisted by PEF was also investigated. Table 1 shows the ingredients, and their elaboration was carried out as follows. First, the shoulder and fat pork meat were minced to 8 mm width by a grinder (M-94-32, GESAME Barcelona, Spain). Then, the minced meat was mixed with the remaining ingredients, and placed in a vacuum kneader (KENADER AVT-50, Gerona, Spain) for 2–3 minutes. Room temperature was always kept under 12 °C. Subsequently, a PEF treatment was applied to the minced meat mixture, which was subsequently stuffed. In order to be able to single out the impact of casing on drying rates, two types of sausage were prepared: one stuffed in natural pork casing, and the other stuffed “without casing” by using gauze to achieve the same shape. For sausages stuffed in natural pork casing (GRACIA GOEZ, S.C.), a sausage stuffer (EM-30, MAINCA Barcelona, Spain) was used. Sausages without casing were manually stuffed. Both varieties of sausages had a diameter of 34–36 mm and a length of 40–50 cm, approximately. All sausages were tied at both ends, dipped in a 5% potassium sorbate solution, and hung on hangers for drying, which consisted in two stages. For the first 7 days, sausages were stored at 4 °C and 90% relative humidity. Then, temperature was subsequently raised from 12 to 18 °C, while the relative humidity of the maturing chamber (INKOA SISTEMAS S.L., Erandio, Spain) was reduced from 85 to 75%^19^. To make results comparable, the same procedure was followed starting from a non-PEF treated minced meat mixture. The study was carried out with three replicates of each sample. The followed experimental procedures have been schematically described in Fig. 1.

**Table 1:** Ingredients used for Spanish dry-cured sausage “longaniza” elaboration.

Ingredients	Amount (kg)
Pork shoulder (purchased at a local supermarket)	7
Pork fat (purchased at a local supermarket)	3
Industrial mixture (Prep. Longaniza Aragón, Gracia Goez, S.C, Zaragoza, Spain)	0.6
Starter culture (Microcampa S cd. 40540; La Campana 1800, Lliria, Spain)	0.001
Potassium sorbate (GraciaGóez, S.C) (5% solution)	Inmersion

### PEF treatments

The PEF unit used in this investigation (EPULSUS-PM1-10, ENERGY PULSE SYSTEM, Lisbon, Portugal) is a Marx generator that can apply monopolar square waveform pulses with a frequency up to 200 Hz. The maximum output voltage and current were 10 kV and 180 A, respectively. The pulse width can be modified, ranging from 5 to 200 μs. Actual voltage, current, and pulse duration were measured using a high voltage probe (TEKTRONIX, P6015A, Wilsonville, OR, USA) and a current probe (STANGENES INDUSTRIES Inc. Palo Alto, CA, USA), respectively, connected to an oscilloscope (TEKTRONIX, TDS 220, Wilsonville, OR, USA). For PEF treatments, the pieces of meat were introduced into the PEF treatment chamber ensuring maximum contact with the electrodes and avoiding air bubbles. Meat was treated in a stainless steel cylindrical-parallel-plate-electrode treatment chamber of 2 cm diameter separated by a methacrylate tube, leaving a gap of 2 cm between both electrodes (2 cm × 2 cm). Meat samples of 6–7 g were prepared using a hole puncher of 2 cm of diameter and then, they were introduced into the PEF chamber. For validation treatments, another cylindrical-parallel-plate-electrode treatment chamber with a diameter of 4 cm and a gap of 5 cm was used (4 cm × 5 cm). The PEF parameters investigated were the electric field strength (from 0.1 to 5.0 kV/cm), pulse width (from 10 to 200 µs), number of pulses (variable according to treatment intensity, from 0 to 200) and specific energy (from 0 to 56 kJ/kg). When evaluating the influence of electric field strength and pulse width, the same total specific energy (28 kJ/kg) was applied by varying the number of pulses depending on the energy per pulse applied with each field strength or pulse width. Two (biological) replicates were carried out for each experimental condition.

In addition to the influence of several different PEF treatment parameters, it was also investigated factors related to the matrix such as mincing size and orientation of muscle fibers. In order to study the influence of muscle fiber orientation with respect to the direction of electric field strength, 2 cm × 2 cm cylinders were cut following two fiber directions: parallel to the applied electric field, and perpendicularly. A meat grinder (Q.5152, Qilive, China) was used to study the influence of mincing size on meat drying. Three mincing sizes were prepared: 2.5, 4.0 and 6.8 mm, with a surface/volume ratio of 2.4, 1.5 and 0.8, respectively. Six replicates were carried out for each mincing size.

For all the PEF treatments, the initial temperature of the samples was about 10 °C increasing in 12 °C because of the treatment, and reaching a final temperature of 22 °C. To compares results, non-PEF treated samples at 10 °C were left at room temperature in closed bottles to temper them up to 22 °C. Then all samples, both control and pulsed, were taken to dehydration when they reached a temperature of 24 °C (ambient temperature).

### Meat/product analysis

#### Water holding capacity (WHC)

Water Holding Capacity was determined as an indirect index of muscle cell electroporation. For this purpose, the centrifugation-based technique^20^ was applied. Samples (5–6 g) were treated at several different PEF intensities, subsequently wrapped in sterile gauze, and introduced into a Falcon tube with glass pearls. Tubes were centrifuged at 1300 rpm for 15 min in a centrifuge (MEGAFUGE 1.0 R, Kendro, Germany) at room temperature. After centrifugation, the meat was weighed (Series 5173, Nahita blue, AUXILAB S.L., Navarra, Spain), and WHC was calculated with Eq. (1).

1 $$WHC=100-(\frac{{W}_{i}-{W}_{f}}{{W}_{i}}\cdot 100)$$

where *W*_*i*_ is the initial weight in grams, and *W*_*f*_ is the final weight of the sample in grams.

The same protocol was applied to non-treated samples that were used as a control.

#### Drying curves

In order to evaluate the effect of the investigated parameters on meat-drying rates, drying curves were obtained. PEF and non-PEF treated pieces of meat were wrapped in gauze and hung on a hook inside a plastic container to ensure that drying conditions were as uniform as possible in terms of temperature, are velocity and relative humidity; the plastic container was placed in a cooling room at 4 °C with a 75 ± 5% relative humidity. Samples were weighed along storage time, and weight loss for each time interval was determined. Drying curves were obtained by plotting the weight loss in % (grams/100 grams of initial weight) versus drying time (in hours).

#### Description of drying curves with mathematical models

To fit the drying curves and calculate kinetic parameters, two mathematical models were compared: one based on Fick’s equation^21^ (Eq. (2)) which describes mass diffusion processes; and the Geeraerd, Herremans, and Van Impe (Eq. (3)) model which describes decreasing asymptotic graphics^22^.

2 $$Y={X}_{e}+({X}_{i}-{X}_{e}){e}^{(-k\cdot t)}$$

3 $$Y=\,\log \,10(({10}^{{X}_{i}}-{10}^{{X}_{e}}){e}^{(-{k}_{max}\cdot t)}+{10}^{{X}_{e}})$$

where *Y* is the weight over time (g); *X*_*i*_ is the initial weight (in %); *t* is the drying time (h); *X*_*e*_ is the weight of the sample after an infinite drying time (%); and *k* or *k*_*max*_ is a constant representing the drying rate (h^−1^). The *X*_*e*_ value was calculated by averaging the values of all samples and, as the results were presented in percentages, the *X*_*i*_ value was 100% in all cases.

The goodness of the fits (R2), the Root Mean Square Error, and Bias (B_f_) and Accuracy (A_f_) factors were used as a quantitative means to measure the performance of the different models^23^. The Bias factor (B_f_) indicates by how much, on average, a model over-predicts (bias factor >1) or under-predicts (bias factor <1) the observed data. The Accuracy factor (A_f_) indicates the degree to which the predictions differ from the observed data. Both curve fitting and calculation of these parameters were carried out using MICROSOFT EXCEL software (MICROSOFT, Seattle, WA, USA) and GRAPHPAD PRISM software (GRAPHPAD Software, Inc., San Diego, CA, USA).

### Statistical analyses

GRAPHPAD PRISM software was used for statistical analyses (one-way ANOVA with Tukey post-test and Student t test) (*p* = 0.05). All experiments were performed at least in triplicate. Error bars in the figures correspond to the mean standard deviation.

## Results and Discussion

The application of PEF with the purpose of accelerating mass transfer phenomena might be of great interest for the improvement of meat product production processes, particularly for cured products. As previously described, curing process involves the release of water from the product to the ambient. In this type of process two mechanisms are happening^24^, diffusion phase in which inter-cellular water molecules diffuse into the extracellular space, and transfer phase in which water evaporates from the surface of the product. By applying PEF to meat samples, aqueous pathways would form in muscle PEF-treated-cells which would enhance the diffusion process, accelerating the curing of the meat.

Specifically, the application of PEF to beef muscle has been shown to modify its structure, which becomes more porous compared with untreated samples and leads to an increase in electrical conductivity and purge loss^25^. These results suggest that PEF induces changes in the microstructure and texture of meat, and it has been pointed out that it could potentially be applied to improve tenderness, to decrease ageing time, or to alter functional properties^11,12,26,27^. All these data are indicators that PEF affects the structure of meat, being of great interest for the improvement/acceleration of the drying and curing processes. However, given the scarce amount of information available regarding this particular application, a thorough study could not be implemented without preliminary groundwork. Therefore, it was necessary to carry out a preparatory stage focused on the study of more basic aspects of muscle cell electroporation. In this initial stage, the influence of various PEF parameters on the permeabilization of muscle cells and, thus, on the acceleration of the drying of muscle tissue was studied. Thereby the influence of the orientation of muscle cells with respect to electric field strength, along with the influence of the particle size of chopped meat on the rate of drying of meat treated by PEF was determined. Thanks to these studies, optimal PEF treatment conditions for meat drying were defined. In the second part of this investigation, these treatment conditions were validated at pilot plant scale by producing Spanish dry-cured sausages.

### Electroporation of muscle cells

**Figure 2:**
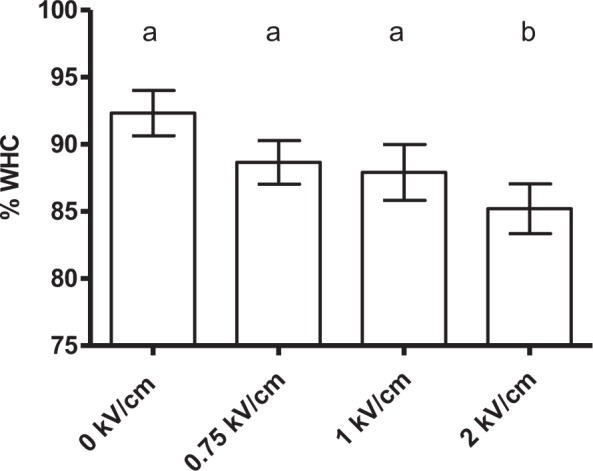
Influence of electric field strength on WHC of pork loin meat. PEF treatment conditions: 28 kJ/kg, 3 µs pulse width, 50 Hz, and perpendicular fiber orientation. The same letter in the upper part of the graph indicates that there are no significant differences (p = 0.05) between the studied variables.

In a first approach, and as an indirect index of cell permeabilization, the WHC of meat was measured. WHC is a parameter closely associated with the amount of immobilized water in the tissues. In order to avoid PSE (Pale, Soft and Exudative) meats that have a lower WHC and might distort results, studies were carried out on pork loins of Duroc breed which is less likely to suffer from such pathological states^28^. Thus, Fig. 2 depicts the influence of electric field strength on the WHC of 2 cm × 2 cm cylindrical meat samples when the same energy level (28 kJ/kg) was applied by pulses of 200 µs at 1 Hz. As observed, the WHC decreased with electric field strength, indicating that cells were electroporated. This also theoretically meant that PEF treatments would facilitate the removal of water in a posterior drying process.

Once the electroporation capacity of PEF in muscle cells was demonstrated, the influence of different parameters related to the product (orientation of muscle fibers, mincing size) and to the PEF treatment (field strength, pulse width, specific energy) on the drying rates of meat was evaluated.

### Influence of the orientation of muscle fibers in meat drying

**Figure 3:**
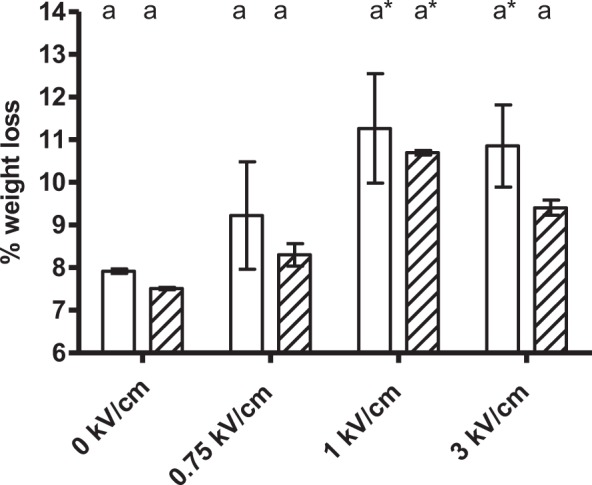
Weight loss value (%) of 2 cm × 2 cm samples of pork loin meat treated by PEF with a perpendicular (white bars) and parallel (stripped bars) orientation of the fibers to the applied electric field strength after 2 h of drying at 4 °C. PEF treatment conditions: 28 kJ/kg, 200 µs pulse width and 1 Hz. The same letter in the upper part of the graph indicates that there are no significant differences (p = 0.05) for the same electric field strength between the samples whose fiber orientation is perpendicular and parallel, and an asterisk indicates significant differences (p = 0.05) between the samples treated with PEF and control samples for the same fiber orientation.

In addition to the technical parameters of the treatment, other factors related to the meat can bear an influence on PEF treatment, such as the orientation of muscle fibers^7^. In order to study this factor, treatments ranging from 0.75 to 3 kV/cm of 200 µs with a total energy of 28 kJ/kg were applied to cylindrical samples of 2 cm × 2 cm placed within the treatment chamber so that muscle fibers were oriented either parallel or perpendicularly to the applied electric field strength. Weight loss after 2 hours of drying at 4 °C was subsequently determined (Fig. 3). As shown in the figure, weight loss increased when PEF treatments were applied, confirming the results displayed in Fig. 2. However, no significant weight loss differences were observed for the samples treated perpendicularly or in parallel to the field strength. The average drying value of the perpendicular samples was nevertheless always greater than that of the samples treated in parallel. This slight effect of muscle cell orientation on PEF electropermeabilization would stand in agreement with the relationship between the transmembrane potential generated in the membrane of a cell considered as a spherical capacitor by an external electric field^29^. The transmembrane potential necessary for the electroporation of a cell depends on the direction of the electric field with respect to the cell’s orientation being lower the transmembrane potential necessary to electroporate cells with perpendicular direction. This hypothesis could explain that the samples treated in a direction perpendicular to the electric field would reach a greater degree of cellular electroporation and therefore incur higher weight losses when the same electric field strength were applied. Based on these results, PEF treatments were applied in subsequent experiments to samples placed within the chamber in such a way that the fibers were oriented perpendicularly to field strength.

### Influence of PEF parameters on meat drying

The evaluation of the influence of PEF treatment parameters on cell permeabilization and, consequently, on drying rates is of great importance in order to optimize the subsequent drying procedures. In this investigation, three parameters were evaluated: electric field strength, specific energy, and pulse width.

**Figure 4:**
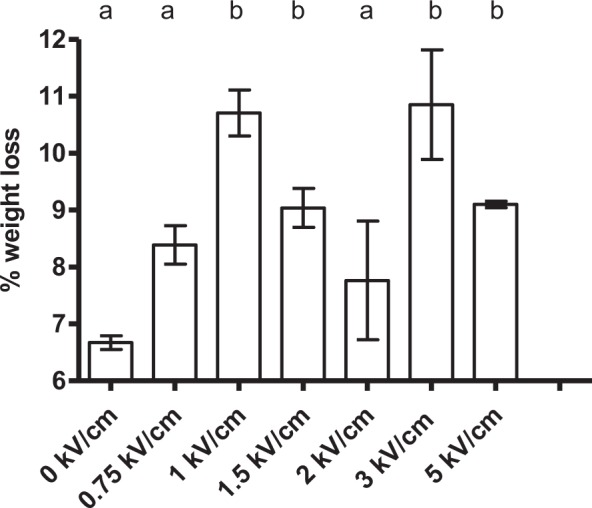
Weight loss value (%) of 2 cm × 2 cm pork loin meat samples after the application of PEF (28 kJ/kg, 200 µs pulse width and 1 Hz) at different electric field strengths (0.75 to 5 kV/cm) and 2 h of drying at 4 °C. The same letter in the upper part of the graph indicates that there are no significant differences (p = 0.05) between the studied variables.

Regarding electric field strength, and as shown in Figs 2 and 3, an increment of electric field strength progressively up to 2 kV/cm proportionately increased the effect of PEF on muscle cells. In order to gain more insight into the relationship between applied electric field strength and the meat drying process, a wider range of electric field strengths was evaluated. Thus, treatments of 0.75, 1.00, 1.50, 2.00, 3.00 and 5.00 kV/cm were applied to cylindrical samples, and weight loss after 2 hours of drying at 4 °C was determined (Fig. 4). For all the evaluated field strengths, the same total specific energy was applied (28 kJ/kg) by varying the number of pulses (of 200 µs). As can be observed in Fig. 4, an increase in water loss of 60.4% (as compared to non-treated samples) was measured when applying 1 kV/cm. The application of greater electric field strengths of up to 5 kV/cm did not result in further weight loss.

These results, indicating that at least 1 kV/cm should be applied to meat tissue in order to observe results, are in agreement with those published in the literature. Khan *et al*.^30^ showed the effect of PEF treatments applied at 1.25 kV/cm (149.8 kJ/kg), but not at 0.31 kV/cm (12.4 kJ/kg) on tenderness and color change of cold-boned beef loins. Faridnia *et al*.^25^ also observed the effects of PEF treatments: it was necessary to apply 1.7 kV/cm (185 kJ/kg; 20 µs) to beef *Biceps femoris* muscle in order to detect variation in its microstructure. In 2001, Gudmundsson and Hafsteinsson^31^ had already indicated that a PEF treatment with a low field strength (1.36 kV/cm and 40 pulses) applied in chicken meat can exert a considerable effect on microstructure by decreasing the size of muscle cells.

**Figure 5:**
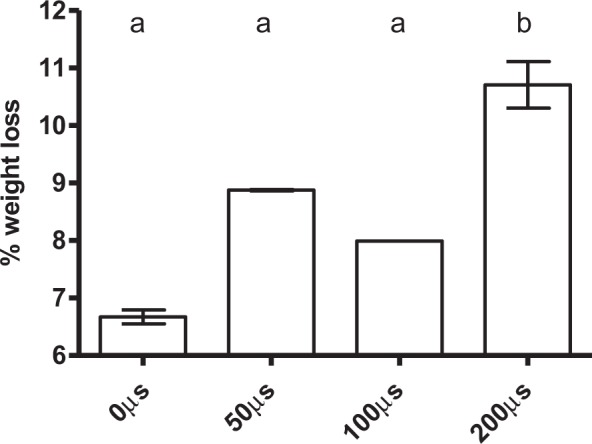
Weight loss value (%) of 2 cm × 2 cm pork loin meat samples after the application of PEF (1 kV/cm, 28 kJ/kg and 1 Hz) at different pulse widths (50 to 200 µs) and 2 h of drying at 4 °C. The same letter in the upper part of the graph indicates that there are no significant differences (p = 0.05) between the studied variables.

Once investigated the effect of field strength on subsequent meat drying, the influence of pulse width was evaluated. Cylindrical samples of 2 cm × 2 cm were treated with several pulses of 1 kV/cm and different pulse widths (from 50 to 200 µs), applying a total specific energy of 28 kJ/kg. Figure 5 shows the influence of pulse width on weight loss after 2 h of drying at 4 °C. As can be observed, weight loss increased when longer pulses (of 1 kV/cm) were applied. Statistically significant differences were observed when pulses of 200 µs were applied. The influence of pulse width has been thoroughly investigated in eukaryotic cells for predominantly medical purposes (evaluating the possibly of obtaining reversible or irreversible electroporation), but it has hardly been evaluated for other fields such as food drying. In the literature, it has been described that longer pulses (in the range of milliseconds) create larger pores which require a longer time interval for resealing compared with pulses in the range of microseconds^32,33^. Our results are in agreement with this general behavior. However, further research needs to be carried out in this field, since it has also been described that in prokaryotic cells shorter pulses of higher field strengths (over 10 kV/cm) were more lethal (produced more irreversible permeabilization) than longer but less intensive pulses^34^. In any case, the influence of pulse width, along with other PEF parameters, might be one of the major causes behind the absence of effects described in the literature after applying PEF treatments to meat, as will be discussed below.

**Figure 6:**
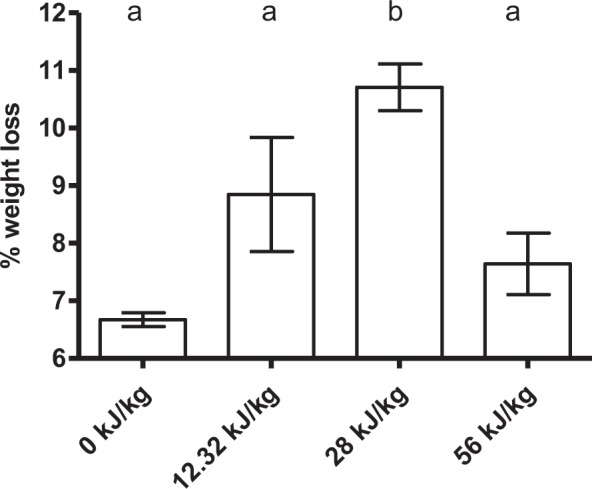
Weight loss value (%) of 2 cm × 2 cm pork loin meat samples after the application of PEF (1 kV/cm, 200 µs pulse width and 1 Hz) at different energy levels (12.3 to 56.0 kJ/kg) and 2 h of drying at 4 °C. The same letter in the upper part of the graph indicates that there are no significant differences (p = 0.05) between the studied variables.

Finally, Fig. 6 shows the influence of specific energy on weight loss of meat cylinders after 2 h of drying at 4 °C when applying a different number of pulses (11, 25 and 50) of 1 kV/cm, 200 µs and 1.12 kJ/kg per pulse. As can be observed, the highest level of drying (37.7%) was observed after the application of 28.0 kJ/kg. When higher energy levels were applied, 56.0 kJ/kg or even higher (data not shown), drying was reduced and samples also presented a “cooked” appearance due to a temperature increase of up to 20 °C, what might have led to protein denaturation. This increase in temperature due to intensive PEF treatments was already observed by Bekhit *et al*.^35^: after applying 68.6–70.8 kJ/kg (10 kV, 0,44–0,48 kV/cm, 20 µs pulse width and 90 Hz) on beef loins, the temperature of the sample raised to 6.5–13.4 °C. Besides, in a preliminary study^11^, they observed that PEF treatments of 10 kV caused intensity cooking effects on the meat edges.

Based on the obtained results, it can be concluded that the application of 25 pulses of 1 kV/cm and 200 µs of pulse width, with a total specific energy of 28 kJ/kg, enabled to improve weight loss of pork loin meat samples in 60% compared to non-treated meat cylinders after 2 h of drying at 4 °C. Other PEF treatment conditions –with different field strengths, pulse widths, and pulse energies – also resulted in improvements of drying rates with respect to control samples, but lower than under optimal conditions. Other investigations have also described weight loss after applying PEF treatments to meat. However, the observed losses were lower, probably due to differences among experimental conditions, including differences in meat cuts and storage conditions, as well as in PEF treatment conditions (e.g. shorter pulses were applied). Thus, O´Dowd *et al*.^36^ observed a purge loss of approximately 1.5% following PEF treatments (2.8 kV/cm; 300 pulses of 20 μs; 225.8 kJ/kg) in a beef semitendinosus muscle treated 72 h post-mortem. Also, a 1.3% increase in weight loss (%), as compared to untreated samples, for 1-day post-mortem beef semimembranosus muscles exposed to PEF (0.27–0.56 kV/cm, 20 μs) and then aged for 3 days, was observed by Bekhit *et al*.^11^. Lower losses were described by Arroyo *et al*.^37^ who observed hardly any weight loss (0.22%) after treating beef *longissimus thoracis* muscle on day 2 post-mortem with up to 600 pulses of 1.4 kV/cm and 20 µs. These losses were increased up to 10% when vacuum-packaged samples were stored for 24 days and were 2% higher than for non-treated samples.

To sum up, it can be concluded from this investigation that, depending on field strength, specific energy and, mainly, pulse width, PEF can produce remarkable effects on meat drying. These data strongly suggest that, as when considering this technology for almost any application, a detailed evaluation of the different PEF processing parameters should be carried out in order to draw meaningful conclusions.

### Influence of sample mincing size on meat drying

**Figure 7:**
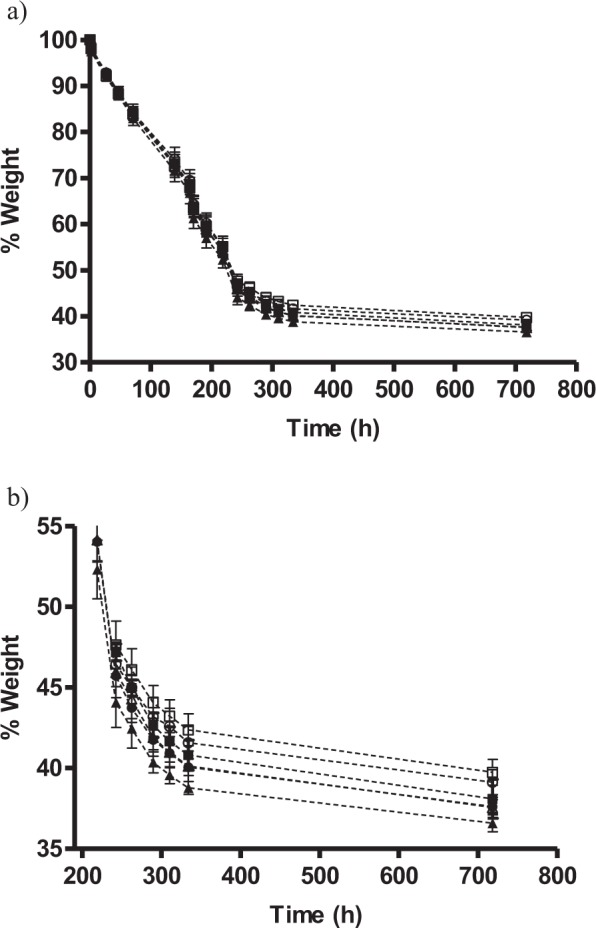
Drying curves (7a) at 4 °C of minced meat samples of 2 cm × 2 cm with different minced sizes, 2.5 (●, ○), 4.0 (▲, △) and 6.8 mm (■, □), for samples treated (close symbols) or not-treated (empty symbols) by PEF (1 kV/cm, 28 kJ/kg, 200 µs pulse width). (**b**) shows a detail of (**a**) after 200 h of drying.

As an intermediate step before studying the potential application of PEF on the elaboration of Spanish dry-cured sausages, the influence of meat mincing size on the subsequent drying process was evaluated. For this purpose, the previously determined treatment (1 kV/cm, 28 kJ/kg, 200 µs) was applied to meat samples of three different mincing sizes (2.5, 4.0, 6.8 mm). Preliminary results indicated that drying was not influenced by the moment at which PEF treatments were applied, e.g., before or after mincing (data not shown). PEF treatments were therefore applied after mincing for simplicity reasons. Figure 7a shows the drying curves (weight loss *versus* time) of both treated and non-PEF treated samples. As can be observed, linear drying kinetics occur for all treatment conditions up to approximately 250 hours, after which drying rates decreased, resulting in hardly 10% further weight reduction in the following 500 hours. In order to more clearly display the differences observed between the different samples, Fig. 7b shows the same drying curves (beginning at 250 hours), but rescaled. As observed, the application of PEF treatments enabled a reduction of the time required to achieve the final drying value after 30 days. Thus, PEF reduced the drying time to 480 (20 days), 580 (approximately, 24 days), and 500 hours (approximately, 21 days) in order to achieve the same weight value (39.1, 37.6, and 39.7%) for mincing sizes of 2.5, 4.0, and 6.8 mm, respectively. That is proportionately equivalent to time reductions ranging from 19 to 33%.

In order to describe in further detail the effect of PEF treatments on the drying process and to evaluate the influence of mincing size, two mathematical models were applied to further describe and analyze the drying curves’ kinetics: the Fick model, and the Geeraerd, Herremans and Van Impe model. Both models accurately described the curves shown in Fig. 7. However, better goodness of the fit parameters (R2 and RMSE) and B_f_ and A_f_ values (Table 2) were obtained with the Geeraerd, Herremans and Van Impe model. That model was therefore chosen to describe the drying curves. Applying it, the drying rate (*k*_max_ value) for each curve, control and PEF treated samples, and the final weight of samples were estimated and then compared (Table 3). As can be deduced from the data presented in the table, PEF-treated samples with a mincing size of 4 mm displayed the highest drying rates (*k*_max_ value) and enabled the obtaining of samples with the lowest weight (*X*_*e*_). For example, the average final weight values observed for control were of 41.4% and, for PEF, 39.9%, resulting in a 3.6% increased drying rate when PEF was applied. The results of the effect of PEF on the drying process are in alignment with those previously mentioned in literature, but in this case slightly higher drying rates and final weights were determined when PEF treatments were applied^11,36,37^. On the other hand, differences in weight values between treated and non-PEF treated samples were smaller when longer drying times (Fig. 7) were evaluated compared with the 2-hour drying tests carried out to prove the effect of PEF parameters (Figs 3–6). This is also in agreement with previously published data^11,36,37^.

**Table 2:** Comparison of the fit of the two mathematical models, Fick model and Geeraerd, Herremans and Van Impe model, used to fit the drying curves of minced pork of different mincing sizes: 2.5 mm, 4.0 mm, 6.8 mm.

Mincing size	Treatment	Model	R²	RMSE	B_f_	A_f_
2.5 mm	Control	Fick	0.9539	4.3552	0,9992	1.0903
		Geeraerd	0.9932	1.7850	1.0011	1.0321
	PEF	Fick	0.9588	4.3035	0.9988	1.0928
		Geeraerd	0.9947	1.6169	1.0011	1.0300
4.0 mm	Control	Fick	0.9602	4.1849	1.0014	1.0868
		Geeraerd	0.9952	1.6474	1.0099	1.0333
	PEF	Fick	0.9595	4.2460	1.0010	1.0925
		Geeraerd	0.9951	1.7195	1.0101	1.0326
6.8 mm	Control	Fick	0.9638	3.7766	0.9958	1.0815
		Geeraerd	0.9954	1.6079	0.9976	1.0297
	PEF	Fick	0.9593	4.1166	0.9976	1.0893
		Geeraerd	0.9950	1.5947	0.9995	1.0307

**Table 3:** Drying parameters calculated using the Geeraerd, Heerremans and Van Impe model to fit the drying curves of minced pork. The same letter indicates that there are no significant differences (p = 0.05) between the studied variables.

	Mincing size (mm)	X_e_ (g/100 g)	k _max_ (h^−1^)
Control	2.5	41.61 ± 0.98^ac^	0.48 ± 0.01^ac^
	4	40.14 ± 0.84^ac^	0.49 ± 0.01^ac^
	6.8	42.35 ± 0.78^a^	0.49 ± 0.01^ac^
PEF	2.5	40.08 ± 0.88^ad^	0.49 ± 0.01^ac^
	4	38.82 ± 0.86^bcd^	0.51 ± 0.01^ab^
	6.8	39.86 ± 0.88^acd^	0.48 ± 0.01^c^

*X*_*e*_: final weight of samples*; k*_*max*_: drying rate.

### PEF-assisted spanish cured sausage drying

**Figure 8:**
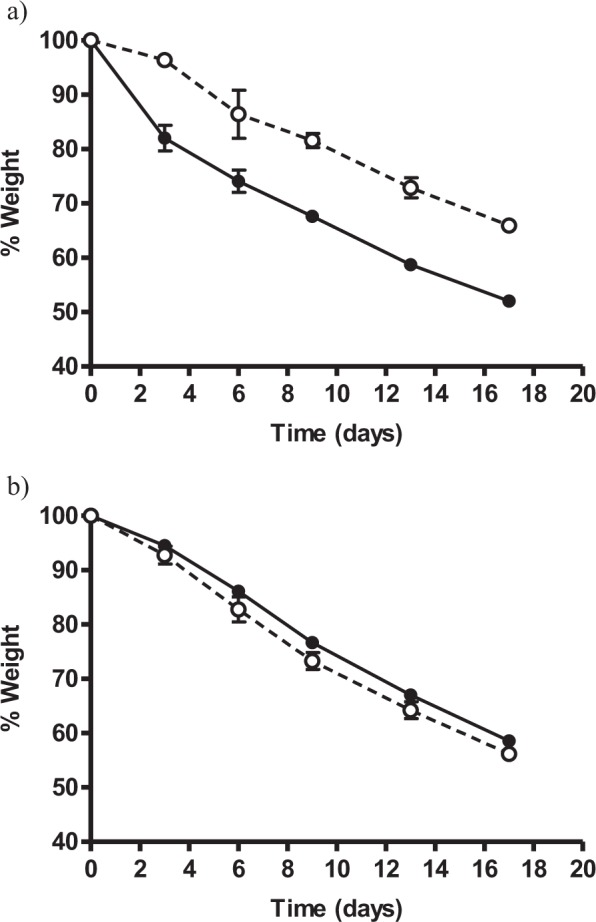
Drying curves of Spanish cured sausages made from treated (1 kV/cm, 200 µs pulse width, 28 kJ/kg) (●) or non-PEF treated meat (○) and stuffed in gauze (**a**) or natural pork casing (**b**) after 17 days of drying in two stages: 7 days at 4 °C and 90% RH; 10 days with an increase in temperature from 12 °C to 18 °C and a reduction of RH from 85 to 75%.

After determining the optimal treatment conditions and evaluating the influence of mincing size at the laboratory level, it was attempted to validate the obtained results by producing a Spanish dry-cured sausage made with PEF-treated meat at pilot plant scale. Under industrial conditions, a cured sausage is stuffed in either natural or artificial casing. Since laboratory optimization tests were performed in this study on meat without casing, and in order to validate the results on a larger scale and to compare the effect of stuffing in a situation more closely resembling the usual practice, sausages were stuffed in gauze simulating the laboratory conditions and in natural pork casing, similarly to an industrial process. Figure 8a,b show the drying curves (sausage weight *versus* time) of both kind of sausages, stuffed in gauze (Fig. 8a) and in natural pork casing (Fig. 8b), prepared from PEF and non-PEF treated meat. As observed, the effect of the PEF treatment on the drying process can be clearly noted in sausages elaborated without casing (Fig. 8a). By the end of the drying process, PEF-treated sausages had 13% lower weight than control samples. Similarly, the application of PEF reduced the drying time required to achieve 66% of original weight (which was the final weight of control samples) from 17 to 9–10 days, which represents a reduction of 41–47%. In the case of cured sausages stuffed in natural pork casings (Fig. 8b), no statistically significant differences were observed between PEF and non-PEF treated samples. These results would indicate that the effect of PEF would be hindered by the casing which would act as a barrier slowing down the water evaporation process. The obtained results demonstrate that the application of PEF treatments to the meat, even when it was minced, led to a considerable reduction of drying time to achieve the same final weight of sausages, thereby confirming the effects previously described at lab scale. Therefore, application of PEF treatments under the conditions here investigated could be used to shorten the drying time of cured-meat products, provided that the casing has adequate water permeability. Further work would be required in order to determine the influence of PEF treatments on the microbiota and the texture of cured meat products.

## Conclusions

In this study, the potential of PEF treatments for the electroporation of muscle cells, leading to a more rapid drying-curing of meat at 4 °C, has been evaluated. It was demonstrated that PEF parameters (electric field strength, pulse width, and specific energy) determine the impact of PEF on meat drying. Optimal conditions for improving the drying of pork loin samples (determined at laboratory scale) were: electric field strength of 1 kV/cm, pulse width of 200 μs, and a total specific energy of 28 kJ/kg. This treatment resulted both in a higher level of drying (60.4%) than control samples, and in a greater drying rate.

Mincing size and muscle cell orientation also bear an influence on the effectiveness of PEF treatments for improving the meat drying process. Thus, perpendicular orientation to the field strength resulted in slightly higher drying values, although non-significant differences were determined, and the fastest drying rates (4%) were observed when PEF was applied to meat grinded into 4 mm particles.

Results obtained at lab scale were validated by preparing Spanish dry-cured sausages at pilot plant scale. A reduction of 41–47% in drying time to achieve the same final weight after treating with PEF (1 kV/cm, 200 µs, 28 kJ/kg) was observed when sausages were dried without casing. However, results suggest that the usefulness of PEF for improving the production process of cured minced meat products might be limited by the water barrier effect exerted by the casing.
